# Prevalence, Bother and Treatment Behavior Related to Lower Urinary Tract Symptoms and Overactive Bladder among Cardiology Patients

**DOI:** 10.3390/jcm9124102

**Published:** 2020-12-19

**Authors:** Mikolaj Przydacz, Przemyslaw Dudek, Piotr Chlosta

**Affiliations:** Department of Urology, Jagiellonian University Medical College, 30-688 Krakow, Poland; przemekdudek@o2.pl (P.D.); piotr.chlosta@gmail.com (P.C.)

**Keywords:** Poland, lower urinary tract symptoms (LUTS), overactive bladder syndrome (OAB), epidemiology, cardiology

## Abstract

Purpose: The aim of this study was to measure, at the population level, the prevalence, bother, and treatment-related behavior for lower urinary tract symptoms (LUTS) and overactive bladder syndrome (OAB) in a large cohort of cardiology patients. Methods: This report is a further analysis of data from LUTS POLAND, a computer-assisted telephone survey that reflected the entire Polish population, stratified by age, sex, and place of residence. LUTS and OAB were assessed by a standardized protocol, the International Continence Society definitions, and validated questionnaires. In addition, all participants provided information regarding their behavior as it related to LUTS treatment. Results: Overall, 6005 participants completed interviews, and 1835 (30.6%) had received treatment by cardiologists. The prevalence of LUTS was 73.3% for cardiology participants compared with 57.0% for respondents who were not treated by cardiologists (*p* < 0.001). There were no differences between men and women in LUTS prevalence for cardiology patients. Nocturia was the most prevalent LUTS. LUTS were often bothersome, and storage symptoms were more bothersome than voiding or postmicturition symptoms. The prevalence of OAB syndrome was 50.7% in cardiology patients, higher than in noncardiology participants (36.6%, *p* < 0.001), and more women were affected than men. Only one-third of cardiology patients who reported LUTS were seeking treatment for LUTS, and most of them received treatment. There were no differences between persons living in urban and rural areas. Conclusions: LUTS and OAB were highly prevalent among cardiology patients. Although LUTS were often bothersome in this unique population, we found that the seeking of treatment for LUTS was minimal. These results highlight the need for cooperation between cardiologists and urologists.

## 1. Introduction

Lower urinary tract symptoms (LUTS) include storage symptoms, voiding symptoms, and postmicturition symptoms [1]. Overactive bladder syndrome (OAB) is a subset of storage symptoms that consists of frequency, urgency, urge urinary incontinence, and nocturia.

LUTS are not sex-specific; both men and women exhibit high prevalence of LUTS and there is an increasing trend with age. Using different survey methodology, data collection, and definition of prevalence, investigators have found LUTS to affect up to 74% of adults aged ≥40 years in Europe and North America [2], 75% in South America [3], and 61% in Asia [4]. In a recent nationwide, population-representative epidemiological study of LUTS in Poland, the first reliable epidemiological study of LUTS in a Central or Eastern Europe country, we reported LUTS prevalence of 69.8% in adults aged ≥40 years; more women were affected than men (72.6% vs. 66.2%) [5].

Notably, LUTS are not disease- or condition-specific. Despite being commonly related to bladder outlet obstruction, LUTS may be indicative of bladder dysfunctions and other structural and/or functional abnormalities of the urinary tract. As well, LUTS may herald many nonurological conditions [6]. Among these conditions, cardiac diseases, in particular, should be considered because, together with LUTS, these dysfunctions are increasingly prevalent because of population aging and comorbid chronic medical conditions. LUTS and cardiac disorders are associated with reduced functional capacity and increased mortality. There has been extensive study of links between cardiovascular disease (CVD) or heart failure (HF) and LUTS [7,8]. Importantly, these relationships may be bidirectional in both men and women because LUTS have been marked as indicators and/or risk factors for predicting future CVD or HF, and CVD or HF have been reported as risk factors for occurrence or worsening of LUTS [9,10,11]. Recent analyses suggest further that therapy for cardiovascular risk reduction might also decrease severity and progression of LUTS [7], whereas worsening CVD or HF either provokes or exacerbates urinary symptoms [12]. Nevertheless, the relationship between LUTS and cardiac disorders may be more complex than one would expect. Although Gacci et al. reported in their systematic review that patients with moderate to severe LUTS had increased risks of major adverse cardiac events [13], a well-designed meta-analysis conducted by Bouwman et al. revealed that the presence of LUTS did not predict CVD in older men who did not have a history of CVD [14].

The pathophysiology underlying the CVD/HF-LUTS relationship is also still under investigation. Yet, important factors that exacerbate or lead to LUTS seems to be metabolic syndrome, chronic inflammation, atherosclerosis-induced pelvic ischemia, impaired nitric-oxide synthase pathway in the endothelium, natriuretic peptides imbalance, concomitant conditions (e.g., diabetes, renal failure, obesity, dyslipidemia), and different medications commonly used by cardiologists (e.g., diuretics, angiotensin-converting enzyme inhibitors, angiotensin receptor blockers, beta-blockers) [7,12,13]. Therefore, LUTS and cardiac disorders share many risk factors.

Several clinic-based studies have shown that some LUTS may be more prevalent in cardiology patients than in the general population [12,15]. However, there is no published population-level study that has evaluated the prevalence, bother, and behavior related to treatment for LUTS, including OAB, in cardiology patients. Further, no population-based study of cardiology patients included the definitions approved by the International Continence Society (ICS). Indeed, experts stipulate that, for high-quality research, investigators should use generally accepted definitions [14]. These data are necessary to promote health, increase awareness, reduce burden of diseases, and attract interdisciplinary frameworks for national health improvement programs. Therefore, the aim of this study was to use standardized definitions provided by the ICS and validated instruments to analyze the prevalence, bother, and treatment-related behavior for LUTS and OAB in a population of Polish cardiology patients.

## 2. Materials and Methods

This study is a further analysis of data from the population-representative cross-sectional epidemiological study LUTS POLAND. Complete details of the study design and methodology are published and presented briefly here [5]. The LUTS POLAND study included representative pools of men and women, aged ≥40 years, living in all geographical regions of Poland (urban and rural areas). The local research ethics committee approved the experiment (1072.6120.160.2019), which is also registered with ClinicalTrials.gov (NCT04121936). All participants provided verbal informed consent.

### 2.1. Study Design

For a survey on population-representative samples in Poland, we decided to use a telephone interview system because face-to-face interviews have limitations in stratifications for place of residence and Internet surveys have limitations in terms of stratifications for age—i.e., older persons are more likely to have poor computer access or they may lack computer skills [16,17]. We used the most recent census and a sample matching technique to create a target sample [18]. We excluded participants with current/past urinary tract infection (within one month) and pregnant women at the time of the survey or women who had given births within the previous six months.

### 2.2. Data Collection

The data collection was conducted by Ipsos Poland, which represented itself with relevant quality certificates (PKJPA, PKJBI, OFBOR, ESOMAR) [19]. All participants reported demographics and presence of LUTS, as suggested by the ICS, which included storage symptoms (frequency, urgency, urgency with fear of leaking, nocturia, urinary incontinence—urge, stress, mixed, leak for no reason), voiding symptoms (intermittency, slow stream, hesitancy, straining, splitting/spraying, terminal dribble), and postmicturition symptoms (incomplete emptying, postmicturition dribble) [1]. Participants used Likert-like scales to rate occurrence of all these symptoms during the previous month (none, less than 1 in 5 times, less than half the time, about half the time, more than half the time, almost always); respondents also rated the bother associated with these symptoms (not at all, a little bit, somewhat, quite a bit, a great deal, a very great deal). The International Prostate Symptom Score (IPSS) [20] and the Overactive Bladder-Validated 8-question Screener (OAB-V8) [21] were also administered; both instruments were completed by men and women. During the telephone survey, respondents also evaluated the impact of bladder problems on treatment seeking, treatment receiving, treatment satisfaction, treatment continuation, and quality of life. Eventually, participants were asked whether they had been treated generally by any healthcare professionals and, if yes, by what type of professionals. All questions, terms, and instruments were validated and presented in Polish. The data analyzed for this paper focus solely on respondents who reported that they received treatment by cardiologists. 

### 2.3. Study Goals

The primary goal of this study was to determine the prevalence of LUTS in a subpopulation of persons treated by cardiologists; the subpopulation was extracted from a large representative pool of Polish adults aged ≥40 years who had responded to a nationwide survey of LUTS. Investigators who have conducted previous large-scale international studies on LUTS prevalence in the general population often used either of the two definitions for LUTS prevalence [2,3,5]. Therefore, to enable comparisons of our findings with all earlier epidemiological analyses of LUTS from the general population, we used both definitions for LUTS prevalence: definition I, symptoms occurring less than half the time or more, and definition II, symptoms occurring half the time or more [2,3]. This approach enabled us to juxtapose the data exclusively for cardiology patients with the data from other studies, typically conducted in the general population.

Secondary study goals included the prevalence of specific LUTS (because of sex differences in LUTS prevalence reported for studies conducted in the general population, we also evaluated specific LUTS separately for men and women treated by cardiologists), the bother of specific LUTS (LUTS were considered bothersome if they were rated at least quite a bit), the prevalence of OAB (score ≥ 8 points from the OAB-V8), overall assessment of severity of LUTS (according to the IPSS), treatment-related behavior for LUTS (treatment seeking, treatment receiving, treatment satisfaction, and treatment continuation), and effect of LUTS on quality of life.

### 2.4. Statistics

Analyses were performed separately for cardiology vs. noncardiology respondents. Further, because of possible sex-based differences in large-scale epidemiological studies that have been conducted in the general population, we also performed separate analyses for cardiology men and cardiology women. Descriptive statistics were used for demographic variables and initial data analysis. The Kruskal–Wallis test was used for continuous variables, and the chi-squared test was used for categorical variables to evaluate differences in LUTS prevalence between the sexes, age groups, and status of cardiology care (i.e., cardiology and noncardiology participants). Regression analysis was also used to investigate the correlations between LUTS/OAB and status of cardiology care regardless of age (a hallmark risk factor for LUTS). Statistical significance was considered to be *p* < 0.05. SPSS Statistics software (IBM Corporation version 24.0, Armonk, NY, USA) was used to conduct data analysis.

In our large LUTS survey, for sample size calculation, we followed the methodology that was used in other studies of the prevalence of LUTS [22]. Therefore, the sample size was calculated based on the population age distribution and expected symptom prevalence [23]. Age standardization depended on the recent census [18]. The sample size calculation with assumption for small statistical error (±1%) estimated an effective sample of 6000 respondents. Because the response rate based on total contacts is typically 25–40% for a nationwide telephone survey, and to reliably calculate poststratification weights, approximately 25,000 contacts were made to obtain the 6000 respondents.

### 2.5. Ethics

The study was performed in compliance with Good Clinical Practice and in accordance with the Declaration of Helsinki. The study was approved by the local research ethics committee.

## 3. Results

Overall, 6005 respondents from throughout Poland participated in the LUTS survey, and 1835 (30.6%) persons reported to have been treated by cardiologists. There were more women (*n* = 1011; mean age 67 ± 10.5) than men (*n* = 824; mean age 66 ± 10.9) in the cardiology group. Of note, the mean age of the cardiology participants was higher than the mean age of noncardiology respondents (67 vs. 58). More cardiology patients lived in urban areas (*n* = 1177) than in rural regions (*n* = 658). Seventy-five percent (*n* = 1383) of the participants reported having at least a secondary education. 

### 3.1. The Prevalence of LUTS

**Figure 1:**
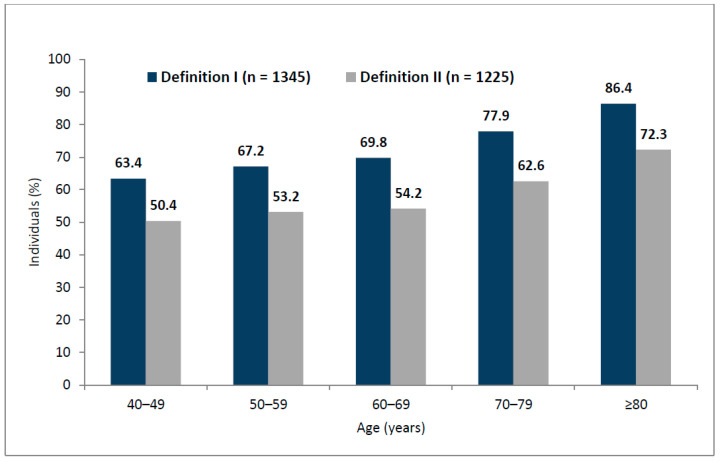
Prevalence of lower urinary tract symptoms based on the two study definitions among cardiology patients (*n* = 1835): definition I—symptoms occurring less than half the time or more; definition II—symptoms occurring about half the time or more.

The prevalence of at least one LUTS at least “less than half the time” (definition I) was 73.3% (*n* = 1345), with no difference between men (*n* = 603; 73.2%) and women (*n* = 742; 73.4%). The prevalence of at least one LUTS at least “half the time” (definition II) was 66.8% (*n* = 1225), with similar prevalence among men (*n* = 550; 66.7%) and women (*n* = 675; 66.8%). Regardless of the LUTS prevalence definition, LUTS were more prevalent in cardiology than noncardiology participants (definition I: 73.3% vs. 57.0%; definition II: 66.8% vs. 49.3%; *p* < 0.001). Importantly, we further found higher prevalence of LUTS in cardiology vs. noncardiology participants independent of age (definition I: relative risk 1.33, 95% confidence interval 1.12–1.65, *p* < 0.01; definition II: relative risk 1.52, 95% confidence interval 1.31–1.74, *p* < 0.001). Regardless of the LUTS prevalence definition, the prevalence of LUTS in cardiology participants increased with age (Figure 1). There were no differences in LUTS prevalence between urban vs. rural status. 

### 3.2. The Prevalence of Specific LUTS

The prevalence of nocturia (definition I: 82.8%; definition II: 46.3%), frequency (definition I: 55.0%; definition II: 38.2%), and urgency (definition I: 25.4%; definition II: 15.3%) were highest among all symptoms in a group of cardiology participants (Table 1). These three storage symptoms were also statistically more prevalent in cardiology vs. noncardiology participants. For the cardiology participants, slow stream was the most frequent voiding symptom and incomplete emptying was the most frequent postmicturition symptom (Table 1). Because LUTS prevalence has a strong age dependency, further age-adjusted analysis for all specific LUTS showed that, regardless of age (*p* < 0.01), nocturia, frequency, urgency, urge urinary incontinence, and straining were more prevalent in cardiology vs. noncardiology participants.

Considering sex differences in the group of cardiology participants, statistically, women more frequently reported frequency, urgency, urgency with fear of leaking and urge, stress, and mixed urinary incontinence compared to men (Table 2). Notably, all these symptoms are storage. Conversely, more men, statistically, reported slow stream, hesitancy, straining, splitting/spraying, and terminal dribble compared with women. Significantly, all these symptoms are voiding. Interestingly, nocturia, the most prevalent symptom, in the population as a whole, was not statistically different between men and women (Table 2). 

### 3.3. The Prevalence of LUTS Subgroups

In the group of cardiology patients, storage symptoms comprised the most prevalent ICS symptom group (definition I: 68.5% overall, 65.9% of men, 70.6% of women; definition II: 63.5% overall; 62% of men, 64.8% of women). Cardiology participants reported voiding symptoms of 32.0% (39.9% of men, 24.5% of women) according to definition I and 21.0% (28.5% of men, 14.9% of women) according to definition II. Postmicturition symptoms had the lowest prevalence (definition I: 15.6% overall, 19.3% of men, 12.6% of women; definition II: 9.0% overall; 10.8% of men; 7.6% of women). 

### 3.4. The Bother of Specific LUTS

The bother rates of specific LUTS were generally comparable between cardiology and noncardiology participants (Table 1). In men treated by cardiologists, leak for no reason and urgency with fear of leaking were the most bothersome symptoms (Table 2). Among women, mixed urinary incontinence and stress urinary incontinence were the most bothersome symptoms (Table 2). Overall, in both cardiology and noncardiology participants (Table 1), as well as men and women treated by cardiologists (Table 2), storage symptoms were more bothersome than voiding or postmicturition symptoms.

### 3.5. The Prevalence of OAB

The prevalence of OAB as measured with the OAB-V8 questionnaire (score ≥ 8 points) was 50.7% in the cardiology group, statistically higher than the 36.6% prevalence in the noncardiology group (*p* < 0.001). OAB was also more common in cardiology vs. noncardiology respondents regardless of age (*p* < 0.001). Considering sex differences among the cardiology participants, OAB was statistically more prevalent in women than in men (54.9% vs. 45.6%, Table 3). In addition, for the cardiology participants, there was a significant correlation for increasing OAB prevalence with increasing age (Table 3).

### 3.6. The Overall Assessment of LUTS Severity with Effects on Quality of Life

With four different IPSS categories (none, mild, moderate, severe), most of our cardiology participants reported mild symptoms (58.9% of men and 66.1% of women, Table 3). The prevalence and severity of symptoms based on the IPSS questionnaire were similar for men and women (*p* = 0.39).

For the question (#8) from the IPSS, “If you were to spend the rest of your life with your urinary condition just the way it is now, how would you feel about that?”, we found that LUTS had a negative impact on quality of life. Among cardiology participants with LUTS at least “less than half the time” (definition I), 30.0% of the respondents were “mixed”, “mostly dissatisfied”, “unhappy”, or “terrible”. Corresponding data for cardiology patients with LUTS occurring “half the time or more” (definition II) were 32.1%.

### 3.7. The Treatment-Related Patterns

#### 3.7.1. Treatment Seeking and Treatment Receiving

One-third (33.3%, *n* = 448) of respondents who were treated by cardiologists and who reported at least one LUTS at least “less than half the time” (definition I) were seeking treatment for their LUTS, and most received treatment (31.9%, *n* = 429). With definition II, i.e., symptoms occurring at least “half the time or more”, 35.0% (*n* = 429) of cardiology respondents were seeking LUTS treatment and most received treatment (33.1%, *n* = 406). Overall, men were more likely than women to seek treatment for their LUTS (definition I: 35.8% vs. 28.7%, *p* < 0.05; definition II: 37.5% vs. 29.6%, *p* < 0.01), but there was no difference between men and women in receiving treatment. In addition, treatment seeking/treatment receiving was independent of urban/rural status. 

#### 3.7.2. Treatment Satisfaction and Treatment Continuation

Most of the cardiology patients who received treatment for their LUTS were satisfied with the treatment (definition I: 77.9%; definition II: 76.8%). However, men were statistically more often satisfied than women (definition I: 84.3% vs. 71.4%, *p* < 0.05; definition II: 84.0% vs. 69.5%, *p* < 0.05). Overall, three out of five cardiology patients who received treatment for their LUTS continued the treatment (definition I: 61.5%; definition II: 62.3%), but men statistically more often continued their LUTS treatment compared with women (definition I: 77.8% vs. 45%, *p* < 0.01; definition II: 79.6% vs. 44.5%, *p* < 0.01).

## 4. Discussion

This study is the first population-based epidemiological investigation that analyzed the prevalence, bother, and behavior related to treatment for LUTS and OAB in an exclusive cohort of cardiology patients. To our knowledge, this cross-sectional study is also the first to use a standardized protocol based on ICS definitions to evaluate cardiology patients who had LUTS. For our analyses, we used reliable data from the population. Our survey covered all geographical regions of the country, including urban and rural areas. In addition, this report is a reference document that will guide future, especially longitudinal, studies to reliably analyze the problem of LUTS and OAB by making use of standardized definitions. Our study supports the recommendations of experts who strongly advocate using generally accepted definitions [14] and include the general population in studies that analyze correlations between LUTS/OAB and cardiology diseases [13]. Considering that the mean age of the worldwide population is steadily increasing, the impact of both LUTS and cardiac disorders on public health is immense, and there is a growing interest in this topic. Hence, monitoring of these disorders is important from the public health perspective [10].

A group of international population-based epidemiological studies has previously estimated the prevalence of LUTS. The Epidemiology Urinary Incontinence and Comorbidities (EPIC) study, a telephone survey in Canada, Germany, Italy, Sweden, and the UK (*n* = 19,165), reported LUTS prevalence of 62.5% in men and 66.6% in women [24]. In Asia, an Internet inquiry with participants from China, Taiwan, and South Korea (*n* = 8284) estimated LUTS prevalence to be 62.8% for men and 59.6% for women [4]. However, all these earlier studies analyzed data from the general population with no separate analyses for participants treated by different healthcare professionals. In our study, we showed that LUTS were highly prevalent in a large cohort of cardiology patients, and more importantly, the prevalence of LUTS was higher in this specific group than for participants without cardiology problems. It is also interesting that, among cardiology participants, we did not observe differences in LUTS prevalence between men and women, although most studies of the general population showed that LUTS are more prevalent in women than in men [2,3,4,24]. The lack of a difference by sex for cardiology individuals may suggest that cardiology-related conditions may lead to LUTS by their own specific sex-independent mechanism. 

In our large and specific cohort of cardiology patients, we also showed that storage symptoms were not the only LUTS in women, because women also reported voiding and postmicturition symptoms. Similarly, voiding and postmicturition symptoms were not the only LUTS in men because men also reported storage symptoms. Although storage symptoms were more prevalent in women than in men, and voiding or postmicturition symptoms were more prevalent in men than in women, the occurrence of different symptoms from different ICS categories in both sexes should compel physicians to perform extensive and thorough diagnostic evaluations with a holistic approach for effective treatment of every cardiology patient. These data may also underline the need for close cooperation between cardiologists and urologists. Urologists should inquire into cardiac disorders in patients with LUTS, and cardiologists should ask about LUTS in cardiology patients, especially for moderate to severe symptoms or those symptoms with high burdens. In addition, a detailed medical history of patient drugs is always needed because LUTS (typically storage symptoms with particular attention to urinary frequency) may be related to medications that cardiologists typically prescribe and which sometimes may be prescribed in doses higher than necessary for optimal control of heart diseases [8]. Further, some management strategies (e.g., fluid control with avoidance of caffeinated beverages, dietary modifications with sodium restriction, physical conditioning with special consideration of pelvic floor muscle exercises, and use of compression stockings) may be beneficial for both cardiac and urological disorders [8]. We should also not overlook the role of primary care physicians, who often coordinate patient therapy between different healthcare professionals. Primary care physicians provide basic care (often sufficient) for many patients with LUTS, and cardiac disorders and follow-up after initiation of any new treatment. Therefore, cooperation between these three types of healthcare providers will promote an integrated approach to treat patients with comorbid LUTS and cardiac disorders.

Special consideration should be given to nocturia, where an individual has to wake at night to void with each void preceded and followed by sleep [1]. Many studies have indicated nocturia as a marker of poor health and clearly associated with various risk factors and comorbidities [25]. There are significant interactions between voiding at night and cardiovascular, metabolic, hormonal, psychiatric, and immunological afflictions that further underline the relationship between nocturia and increased risk of mortality [26]. Among these conditions, cardiac diseases should be especially considered because nocturia is one of the symptoms of heart failure currently characterized as a pandemic that affects at least 26 million people [27]. The main mechanisms involved in nocturia in heart diseases are renal hyperfiltration and an increase in atrial natriuretic peptide (typically in congestive heart failure) [28]. The use of calcium antagonists (e.g., amlodipine) may be further associated with peripheral edema, and the nightly reabsorption of peripheral edema increases urine volume. In addition, beta-blockers decrease bladder capacity and can cause nocturia if taken just before going to bed. Night diuretics may also exacerbate nocturia with increased urine production. In our study, nocturia was the most prevalent symptom that affected more than 80% of cardiology respondents with higher prevalence in cardiology vs. noncardiology participants. Therefore, our results support a broad, symptom-driven approach to LUTS because LUTS are neither disease- nor condition-specific, and they may herald many nonurological conditions [6]. 

OAB is defined as a combination of symptoms that may represent OAB or coexistent conditions. The diagnosis of OAB can be established in the absence of urinary tract infection or another obvious pathology. With the OAB-V8 questionnaire, a validated screening tool for OAB, more than 50% of our cardiology respondents met the threshold for possible OAB diagnosis. Importantly, in our study, statistically more cardiology than noncardiology respondents met this threshold. Clinicians should devote attention to this correlation because a link between OAB and cardiac diseases has been reported. Kilinc et al. showed that the incidence of severe coronary artery disease was higher in patients with OAB symptoms [29]. The authors explained that atherosclerosis, a systemic disease, affects several vessels, including coronary and pelvic arteries, and a close relationship between pelvic ischemia and LUTS, including OAB, is well-known [30]. Pelvic ischemia may also trigger other LUTS and may be related to cardiac ischemia. Because some urological disorders, such as erectile dysfunction, have been indicated as signs of ischemic coronary disease or other serious cardiac pathologies, our results seem to support the hypothesis that OAB may be a red flag for cardiology disorders [11,31]. Kupelian et al., in their prospective well-designed longitudinal study, found that symptoms of a storage type typical of OAB may act as sentinel symptoms of increased cardiometabolic risk, which provides an opportunity for early intervention and assessment of disease risk [11]. Chiu et al. found that patients with HF had more storage urinary symptoms suggestive of OAB compared with age-matched controls; in addition, poor heart function correlated with the worst OAB symptoms [32]. Other studies underlined the negative effects of antidiuretics, particularly the loop-type commonly used by cardiology patients on the increased prevalence of OAB; these drugs may increase urinary frequency and may cause urinary urgency and incontinence, especially in the older population [33]. Therefore, urologists should carefully evaluate their OAB patients and, in a case of doubt, they should refer their patients to a cardiologist. 

Numerous studies have shown that LUTS may be highly bothersome and, therefore, incline people to seek treatment. We demonstrated that storage symptoms were more bothersome than voiding or postmicturition symptoms in cardiology participants, with symptoms of urinary incontinence being the most bothersome. Participants with LUTS also often had concerns about their urinary-specific quality of life. However, only one-third of cardiology patients reporting LUTS sought treatment for their LUTS. Notably, all these results are consistent with findings from other studies conducted in the general population [4,24]. For instance, the Epidemiology of Lower Urinary Tract Symptoms (EpiLUTS) study, an Internet-based population inquiry in Sweden, the USA, and the UK, determined that only 29% of men and 28% of women with LUTS sought treatment for their urinary symptoms [34]. Therefore, the low rate of healthcare seeking related to LUTS is a significant concern regardless of cardiology care. These findings may also suggest that there is a need to improve the awareness of coexistence of LUTS, including OAB and cardiac disorders. The lack of knowledge of both patients and physicians may present barriers to healthcare seeking and treatment receiving. Possible impediments to patients seeking LUTS treatment may include embarrassment, concern about taking another medication, cost, the belief that there is no effective treatment, and fear of surgery [15]. In addition, LUTS are not simply and routinely prioritized in discussions regarding cardiac management. This situation is yet another reason for close cooperation between cardiologists and urologists. Because cardiac disorders may explain the increased prevalence of LUTS in this unique population, common urological illnesses (e.g., benign prostatic hyperplasia, urinary tract infections) may further exacerbate reported LUTS or be the main reason for LUTS presence. 

A strong point of our study was a large sample size with well-balanced demographic characteristics. We stratified the variables by the recent census to ensure adequate representation of the population. In contrast to clinic-based analyses, our study design with data from the population level contributed to decreasing the selection bias. We included a variety of questions in the survey, and we employed well-established and validated diagnostic tools. Although we used validated questionnaires, in fact, there is no gold standard instrument for the overall assessment of LUTS at the population level. Although the IPSS is the most used questionnaire for LUTS assessment, it is limited by inclusion of only seven questions, and the assessment of storage LUTS is particularly restricted. The OAB-V8 questionnaire is a screening tool for OAB that evaluates only storage symptoms; the instrument does not investigate any voiding or postmicturition symptoms. Therefore, even if investigators use validated reliable questionnaires, LUTS prevalence should still be analyzed based on the definitions provided by the International Continence Society [22]. We defined the presence of LUTS with two definitions that have been used in contemporary epidemiological studies that analyzed LUTS prevalence in the general population [2,3,5]. Thus, this two-definition approach enabled us to analyze LUTS prevalence based on the classifications currently recognized by the International Continence Society. However, our study was not free from limitations. This study was cross-sectional and future longitudinal data are needed to clarify the relationships between LUTS and cardiac disorders, as suggested by Bouwman et al. [35]. We used self-reports without medical evaluation to measure LUTS, and we relied on telephone interviews during which some individuals may not have provided accurate answers (especially with intimate information such as urinary incontinence). We also used questionnaires that were designed originally for self-completion (i.e., IPSS and OAB-V8). The use of these questionnaires could have led to interviewer bias, although the interviewers did not substantively aid participants in completion of the questions. In addition, we did not ask for specific concomitant cardiology conditions. Nevertheless, without clinical verification, these pieces of information would be difficult to obtain reliably from a self-reporting participant during a telephone interview. Coyne et al. have described this significant information bias of population-based self-report data [36]. In this unique population, patients often present with a combination of multiple disease or conditions, which are influenced by multiple factors.

## 5. Conclusions

This study is the first that analyzed the prevalence, bother, and behavior related to treatment for LUTS and OAB in a large cohort of cardiology patients at the population level. LUTS were highly prevalent in cardiology patients and more common than in respondents without cardiology concerns. Specific symptoms and symptom groups were not attributed to only men or to only women. LUTS were often bothersome and had negative effects on quality of life. Eventually, in this exclusive cohort, the degree of treatment seeking for LUTS was low. Therefore, it may be important for clinicians to inquire for the presence of LUTS and OAB in cardiology patients and to incorporate appropriate interventions or refer patients to urologists or primary care physicians. Having identified the prevalence and true burden of LUTS and the low seeking rate for LUTS therapy of cardiology patients, we can begin to develop strategies to specifically address these problems, while optimizing medication adherence, overall management, and close cooperation between diverse healthcare professionals.

## Figures and Tables

**Table 1:** Prevalence of specific symptoms according to definition I (symptoms occurring less than half the time or more) and definition II (symptoms occurring about half the time or more) and associated bother in cardiology and noncardiology participants. The bold numbers increase the visibility of the statistically different data.

	Cardiology Participants (*n* = 1835)						Noncardiology Participants (*n* = 4170)					
	Symptom Prevalence (Definition I)		Symptom Prevalence (Definition II)		Prevalence of Bother (at Least Quite a Bit) ^a^		Symptom Prevalence (Definition I)		Symptom Prevalence (Definition II)		Prevalence of Bother (at Least Quite a Bit) ^a^	
	*n*	%	*n*	%	*n*	%	*n*	%	*n*	%	*n*	%
Storage symptoms												
Nocturia ^b^	1520	82.8 ***	849	46.3 **	401	47.2	2907	**69.7**	1319	**31.6**	581	44.0
Frequency	1009	55.0 ***	701	38.2 **	373	53.2	1362	**32.7**	996	**23.9**	504	50.6
Urgency	467	25.4 **	281	15.3 *	233	82.9	704	**16.9**	392	**9.4**	320	81.6
Urgency with fear of leaking	293	16.0 *	217	11.8 *	188	86.6	446	**10.7**	294	**7.1**	250	85.0
Urge urinary incontinence	191	10.4 *	104	5.7	87	83.7	234	**5.6**	138	3.3	122	88.4
Stress urinary incontinence	190	10.4	119	6.5 *	108	90.8	278	6.7	160	**3.8**	149	93.1
Mixed urinary incontinence ^c^	98	5.3	49	2.7	45	91.8	132	3.2	74	1.8	68	91.9
Leak for no reason	99	5.4	57	3.1	52	91.2	128	3.1	66	1.6	57	86.4
Voiding symptoms												
Intermittency	240	13.1	153	8.3 *	102	66.7	299	7.2	173	**4.1**	110	63.6
Slow stream	340	18.5 *	189	10.3 *	116	61.4	439	**10.5**	245	**5.9**	138	56.3
Hesitancy	183	10.0	96	5.2	81	84.4 *	258	6.2	117	2.8	76	**65.0**
Straining	128	7.0 *	67	3.7 *	54	80.6	140	**3.4**	73	**1.8**	59	80.8
Splitting/spraying	183	10.0	93	5.1	64	68.8	244	5.9	119	2.9	71	59.7
Terminal dribble	336	18.3	215	11.7	133	61.9	560	13.4	331	7.9	169	51.1
Postmicturition symptoms												
Incomplete emptying	229	12.5	136	7.4	103	75.7	347	8.3	191	4.6	136	71.2
Postmicturition dribble	121	6.6	61	3.3	53	86.9	185	4.4	94	2.3	74	78.7

^a^ Prevalence of bother was based on definition II; ^b^ Nocturia was defined as two or more voids per night; ^c^ Participants who reported both urge and stress urinary incontinence symptoms were classified as having mixed urinary incontinence; * *p* ≤ 0.05, cardiology vs. noncardiology participants; ** *p* ≤ 0.01, cardiology vs. noncardiology participants; *** *p* ≤ 0.001, cardiology vs. noncardiology participants.

**Table 2:** Prevalence of specific symptoms according to definition I (symptoms occurring less than half the time or more) and definition II (symptoms occurring about half the time or more) and associated bother in men and women treated by a cardiologist. The bold numbers increase the visibility of the statistically different data.

	Men (*n* = 824)						Women (*n* = 1011)					
	Symptom Prevalence (Definition I)		Symptom Prevalence (Definition II)		Prevalence of Bother (at Least Quite a Bit) ^a^		Symptom Prevalence (Definition I)		Symptom Prevalence (Definition II)		Prevalence of Bother (at Least Quite a Bit) ^a^	
	*n*	%	*n*	%	*n*	%	*n*	%	*n*	%	*n*	%
Storage symptoms												
Nocturia ^b^	681	82.6	383	46.5	177	46.2	839	83.0	466	46.1	224	48.1
Frequency	404	49.0 **	341	41.4 *	162	47.5 *	605	**59.8**	366	**36.2**	211	**57.7**
Urgency	185	22.5 *	101	12.3 *	80	79.2	282	**27.9**	180	**17.8**	153	85.0
Urgency with fear of leaking	109	13.2 *	78	9.5	73	93.6	184	**18.2**	139	13.7	115	82.7
Urge urinary incontinence	49	5.9 **	25	3.0 *	19	76.0	142	**14.0**	79	**7.8**	68	86.1
Stress urinary incontinence	22	2.7 ***	14	1.7 ***	12	85.7	168	**16.6**	105	**10.4**	96	91.4
Mixed urinary incontinence ^c^	13	1.6 ***	7	0.8 ***	6	85.7	85	**8.4**	42	**4.2**	39	92.9
Leak for no reason	33	4.0	15	1.8	14	93.3	66	6.5	42	4.2	38	90.5
Voiding symptoms												
Intermittency	136	16.9	89	10.8	58	65.2	104	10.3	64	6.3	44	68.8
Slow stream	209	25.4 *	119	14.4 *	75	63.0	131	**13.0**	70	**6.9**	41	58.6
Hesitancy	122	14.8 **	65	7.9 *	55	84.6	61	**6.0**	31	**3.1**	26	83.9
Straining	90	10.9 **	51	6.2 **	43	84.3 *	38	**3.8**	16	**1.6**	11	**68.8**
Splitting/spraying	122	14.8 **	61	7.4 **	40	65.6	61	**6.0**	32	**3.2**	24	75.0
Terminal dribble	210	25.5 **	144	17.5 **	88	61.1	126	**12.5**	71	**7.0**	45	63.4
Postmicturition symptoms												
Incomplete emptying	133	16.1 *	75	9.1	60	80.0	96	**9.5**	61	6.0	43	70.5
Postmicturition dribble	67	8.1	34	4.1	30	88.2	54	5.3	27	2.7	23	85.2

^a^ Prevalence of bother was based on definition II; ^b^ Nocturia was defined as two or more voids per night; ^c^ Participants who reported both urge and stress urinary incontinence symptoms were classified as having mixed urinary incontinence; * *p* ≤ 0.05, men vs. women; ** *p* ≤ 0.01, men vs. women; *** *p* ≤ 0.001, men vs. women.

**Table 3:** Data from the Overactive Bladder-Validated 8-question Screener (OAB-V8) (prevalence of overactive bladder syndrome (OAB)) and the International Prostate Symptom Score (IPSS) (prevalence and severity of lower urinary tract symptoms (LUTS)) questionnaires completed by men and women treated by cardiologists.

	Sex						
	Men		Women		Total		*p* Value
	*n*	%	*n*	%	*n*	%	
OAB-V8							
OAB-V8 score ≥ 8 (cardiology participants)	376	45.6	555	54.9	931	50.7	<0.01
Age category							<0.01
40–49	19	27.9	19	34.5	38	30.9	
50–59	65	42.5	88	53.7	153	48.3	
60–69	136	46.7	187	53.0	323	50.2	
70–79	96	43.0	185	58.4	281	52.0	
≥80	60	67.4	76	62.3	136	64.5	
IPSS							
Category (defined by the IPSS score)	824	100	1011	100	1835	100	*p* = 0.39
None (score 0)	52	6.3	66	6.5	118	6.4	
Mild (score 1–7)	485	58.9	668	66.1	1153	62.8	
Moderate (score 8–19)	241	29.2	254	25.1	495	27.0	
Severe (score 20–35)	46	5.6	23	2.3	69	3.8	
